# Important progress towards elimination of onchocerciasis in the West Region of Cameroon

**DOI:** 10.1186/s13071-017-2301-7

**Published:** 2017-08-03

**Authors:** Guy-Roger Kamga, Fanny N. Dissak-Delon, Hugues C. Nana-Djeunga, Benjamin D. Biholong, Stephen Mbigha Ghogomu, Jacob Souopgui, Joseph Kamgno, Annie Robert

**Affiliations:** 1 Ministry of Public Health, N°8, Rue 3038 quartier du Lac, Yaoundé, Cameroon; 2 Centre for Research on Filariasis and other Tropical Diseases (CRFilMT), P.O. Box 5797, Yaoundé, Cameroon; 3 Institut de Recherche Expérimentale et Clinique, Faculté de santé publique, Université catholique de Louvain, Clos Chapelle-aux-champs 30 bte B1.30.13, BE-1200 Brussels, Belgium; 4 Institute of Biology and Molecular Medicine, Rue des professeurs Jeener et Brachet 12 BE-6041 Gosselies, Université Libre de Bruxelles, Brussels, Belgium; 5 Faculty of Medicine and Biomedical Sciences, University of Yaoundé 1, P.O. Box 1364, Yaoundé, Cameroon; 6 Molecular and Cell Biology Laboratory, Department of Biochemistry and Molecular Biology, University of Buea, P.O. Box 63, Buea, Cameroon

**Keywords:** Onchocerciasis, Ivermectin, Persistence, Elimination, West Region, Cameroon

## Abstract

**Background:**

After more than a decade of community-directed treatment with ivermectin (CDTI) in the West Region of Cameroon, epidemiological evaluation conducted in 2011 showed that onchocerciasis endemicity was still high in some communities. The conceptual framework for onchocerciasis elimination recommends in such case, to conduct additional phase 1A surveys at intervals of three to four years. Therefore, to assess the progress made towards the elimination of onchocerciasis in the West CDTI projects, we conducted a cross-sectional survey in May 2015 in 15 unevaluated communities where the highest baseline endemicity level were found in 1996. All volunteers living for at least five years in the community, aged five years or more, underwent clinical and parasitological examinations. Individual adherence to ivermectin treatment was also assessed. Analyses of data were weighted proportionally to age and gender distribution in the population.

**Results:**

The mean age was 28.4 ± 22.2 years and there were 55% of women among the 2058 individuals examined. The weighted prevalences were 5.5%, 2.1% and 1.7% for microfilaridermia, nodule and cutaneous signs, respectively. The weighted microfilaridermia prevalences varied from 4.0 in 5–9 years old to 11.6% in 40–49 years old. In the 30 children under 10 years examined in Makouopsap, the weighted prevalences were 49.9% for microfilaridermia and 13.3% for nodule. In surveyed communities, the weighted prevalences varied from 0 to 41.6% for microfilaridermia, with 11 (73.3%) communities having <5%. Except Makouopsap which had 41.6%, all the surveyed communities were below 15% for microfilaridermia prevalence. The community microfilarial load (CMFL) expressed in microfilariae/skin snip (mf/ss), also significantly dropped by 98–100%, from 3.75–33.16 mf/ss in 1996 to 0–0.94 mf/ss in 2015. The weighted therapeutic coverage in 2014 was 69.4% and the 5 years’ adherence was only 39.3% among participants.

**Conclusions:**

After more than 15 years of CDTI, there is an important progress towards the elimination of onchocerciasis in the communities surveyed. Innovative strategy like semi-annual ivermectin treatment plus vector control or the adjunction of a vector control strategy to the current annual treatment should be implemented in the bordering districts of the Centre and West Regions, as well as in other parts of the country with persistent high prevalences in the sight of onchocerciasis elimination.

**Electronic supplementary material:**

The online version of this article (doi:10.1186/s13071-017-2301-7) contains supplementary material, which is available to authorized users.

## Background

Onchocerciasis, better known as river blindness, is a debilitating insect-borne parasitic disease caused by *Onchocerca volvulus* and transmitted via the bites of blackflies of the genus *Simulium*. The larvae and pupae of the latter develop in fast-flowing and well-oxygenated streams and rivers. The prevalence of infection and disease in a community is therefore related to riverine breeding sites of the vector. The disease is endemic in 31 African countries, in Yemen and in two localized foci of two Latin American countries (Brazil and Venezuela). It was eliminated in Columbia (2013), Ecuador (2014), Mexico (2015) and Guatemala (2016) [1]. Among the estimated 187 million people living in areas where there is a potential for transmission of the parasite worldwide in 2015, 99% of them live in Africa. It is estimated that 37 million people were infected in 1995, when the African Programme for Onchocerciasis Control (APOC) was launched [2, 3].

The adult *O. volvulus* has an estimated average lifespan of 10 years with some worms thought to live as long as 15 years [4, 5]. The female adult worm produces thousands of microfilariae daily which invade the host dermis and eyes. The various skin symptoms (severe skin damage with unrelenting itches) and ocular symptoms (visual impairment and blindness) of onchocerciasis [6–8] are induced by the inflammatory responses to the dead of microfilariae [9]. After trachoma, irreversible onchocercal blindness is ranked as the world’s second leading infectious cause of preventable blindness [10]. Onchocerciasis is a neglected tropical disease (NTD), that creates stigma, generates and perpetuates poverty, and is an impediment to socioeconomic development.

Ivermectin is currently the only known effective and safe drug used for mass treatments against onchocerciasis. However, it has a limited macrofilaricidal activity, thus treatments must be repeated for at least 12–15 years, corresponding to the reproductive lifespan of the adult worm when exposed to drug pressure. The control of onchocerciasis has been quite successful with the implementation of Community Directed Treatment with Ivermectin (CDTI). This strategy, proposed by the World Health Organization (WHO) through APOC, has significantly improved ivermectin treatment coverage [11–13]. While this drug has been administrated twice or four times a year in the small and well-delineated endemic communities of the Americas, only single doses have been given yearly in the much larger foci of African endemic countries [14, 15]. As a consequence, the programmes in the Americas are highly successful and the disease has been eliminated in four Latin America countries [16–19], while it remains a public health problem in Africa. Nevertheless, new evidence points exist towards the possibility of successful elimination of river blindness in Africa using ivermectin solely [20, 21]. Indeed, a spectacular decrease in microfilaridermia prevalence below 1% was reported in Mali, Senegal and Uganda [21, 22], as well as in some CDTI-projects in the APOC countries [1, 23, 24]. However, despite more than 20 years of disease control, onchocerciasis remains a major concern in some foci of several endemic countries, including Cameroon [24, 25].

The phase 1 of the APOC’s conceptual framework for onchocerciasis elimination [26] has two sequential objectives: (i) to evaluate the progress towards elimination by assessing the decline in infection levels in the human population towards provisional thresholds for elimination (evaluation phase 1A), and (ii) to confirm, using both parasitological and entomological indicators, that the provisional threshold has been reached and that the treatment can be safely stopped (evaluation phase 1B and the end of phase 1).

Indeed, recent phase 1A surveys conducted in 2011 by WHO/APOC revealed onchocerciasis prevalences above 60% in some foci of the Centre 1, Littoral 2 and West CDTI-projects in Cameroon [24]. The reasons for such persistence of the infection are to be elucidated yet, and it is not clear whether this is due to low treatment coverage, systematic non-adherence of a proportion of the population, or suboptimal response of the parasite to ivermectin. These poor results led to the implementation of some corrective measures (frequent and regular supportive supervision, data quality audit, community self-monitoring and a better drug management) by the National Onchocerciasis Control Programme (NOCP) to improve the CDTI performances. In such case where the prevalence remains high, additional phase 1A surveys would be needed at intervals of three to four years.

The objective of the present survey was therefore, to assess the progress made towards the elimination of onchocerciasis in the West CDTI project by assessing unevaluated communities where the highest endemicity levels were found in 1996, before the launch of control measures. This assessment was based on previous APOC’s conceptual framework for onchocerciasis elimination as it was conducted before the publication of the new 2016 WHO revised guidelines on stopping mass drug administration (MDA) and verifying elimination which advocate xenomonitoring and serology [27].

## Methods

### Study area

**Fig. 1:**
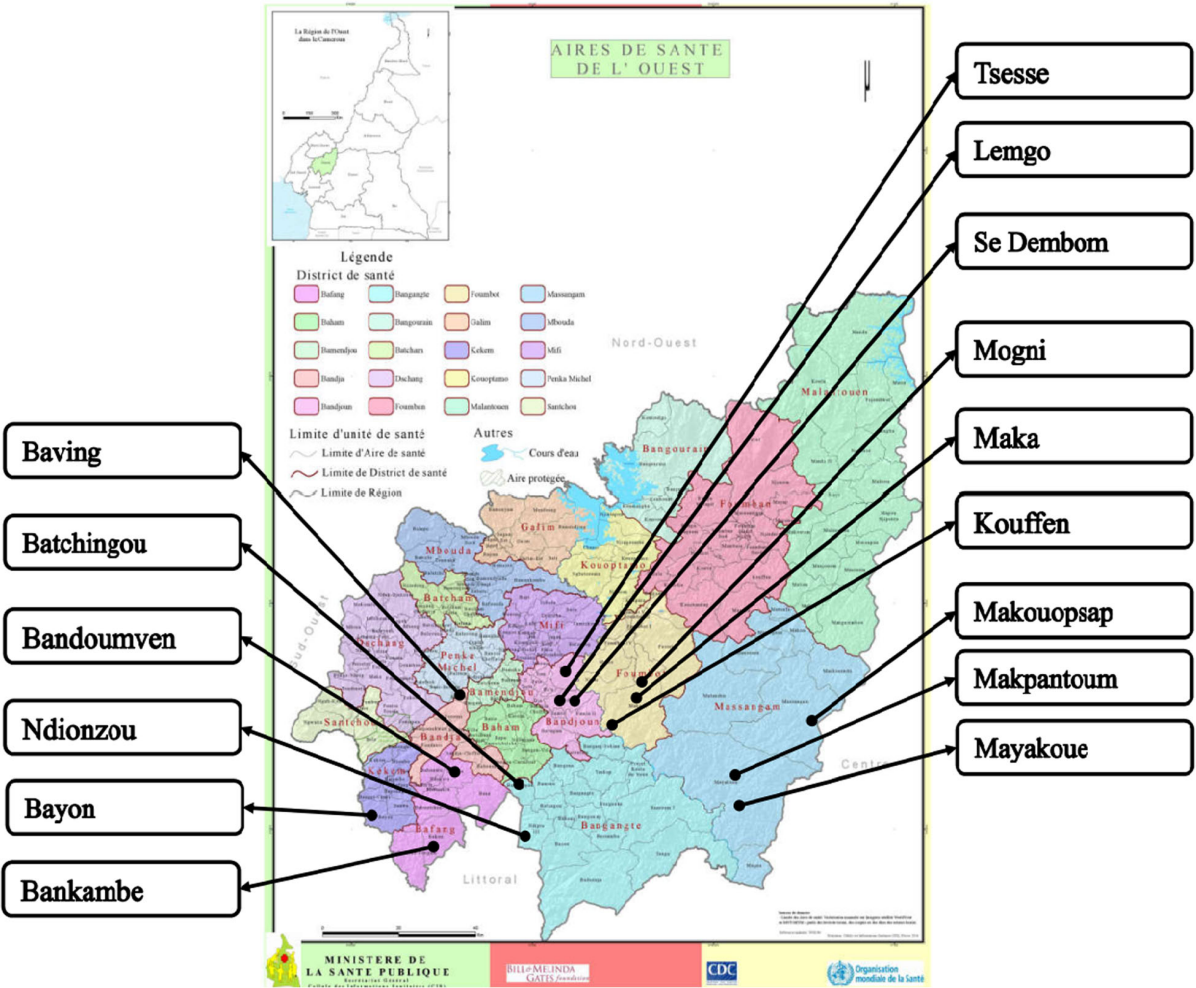
Map of the West Region of Cameroon showing the surveyed communities

The present study was conducted in seven health districts (HD) out of the 20 of the West Region. The West Region (5°30′0″N, 10°30′0″E) is 13,892 km^2^ of territory located in the central-western portion of the Republic of Cameroon. It borders the Northwest Region to the northwest, the Adamawa Region to the northeast, the Centre Region to the southeast, the Littoral Region to the southwest, and the Southwest Region to the west (Fig. 1). The West Region is the smallest of Cameroon’s ten regions in area. However, with its population estimated at 1,895,102 inhabitants in 2014 based on a census conducted by community directed distributors (CDD), it has the highest population density (136.4 inhabitants/km^2^). Its relief is mountainous with altitude varying from as low as 500 m in the Noun and Nkam valleys to 1000–2500 m in Western High Plateau. It is a forest-savanna transition zone, irrigated by many fast-flowing rivers including the tributaries of the Sanaga in the east (Métchié, Mifi, Nkoup, Ndé, Noun) and the tributaries of the Nkam in the south (Ménoua, Makombé, Ngoum and Mwanké) that support breeding of blackfly which transmit onchocerciasis throughout the year. The climate is equatorial of the Cameroon sub-variety in the northwestern third and equatorial of the Guinea type in the southeastern two-thirds, with a long rainy season (March-October) and a short dry season (November-February). Rainfall, moderated by the mountains, averages 1000–2000 mm per year. The West is one of the Cameroon’s richest economic areas due primarily to its agricultural prosperity and the enterprising traditions of the Bamileke people, accounting for 90% of the total population.

### Selection of communities

Surveyed communities were selected according to the availability of 1996 baseline data [28] and the results of the 2011 follow-up survey [29]. In 1996, a baseline survey was conducted in 38 communities belonging to six HD at that time, 30 of them being mesoendemic (35% ≤ prevalence ˂ 60%) or hyperendemic (prevalence ≥ 60%). Among the later, 12 communities were selected and evaluated in follow-up surveys in 2005, 2006 or 2011. From unevaluated communities, we selected 13 presenting the highest microfilaridermia prevalence and/or community microfilarial load (CMFL) distributed throughout the region and belonging to seven HD as follows: Bafang (2); Bandja (1); Bandjoun (3); Bangangté (2); Foumbot (3); Kékem (1); Massangam (1). We added the community Makouopsap that was evaluated only in 2011, and showed a high prevalence (60%), and the community Makpantoum, never evaluated, both belonging to the Massangam HD (Table 1; Fig. 1).

**Table 1:** Baseline data in selected communities

Health district	Community	Kamgno 1996 (baseline data) [28]					Katabarwa 2011 [29]			
		*N1*	Weighted nodule prevalence (%)	*N2*	Weighted mf prevalence (%)	CMFL (mf/ss)	*N1*	Crude nodule prevalence (%)	*N2*	Crude mf prevalence (%)
Bafang	Bandoumven	29	51.7	64	65.1	5.79				
	Bankambe	25	64.0	122	75.8	3.75				
Bandja	Baving	31	35.5	45	75.3	7.10				
Bandjoun	Lemgo	28	71.4	43	85.5	13.19				
	Sé-Dembom	32	81.3	51	82.4	26.69				
	Tsessè	29	75.9	42	85.6	13.18				
Bangangté	Batchingou	29	75.9	84	72.3	7.67	247	11.7	247	18.2
	Ndionzou	24	95.8	107	90.2	7.90				
Foumbot	Kouffen	27	88.9	33	81.8	19.17				
	Maka	30	76.7	53	76.9	20.16				
	Mogni	29	79.3	38	78.3	33.16				
Kékem	Bayon	27	55.6	127	83.5	6.79				
Massangam	Makouopsap	nd	nd	nd	nd	nd	99	43.4	99	59.6
	Makpantoum	nd	nd	nd	nd	nd	nd	nd	nd	nd
	Mayakoué	25	88.0	61	91.1	18.76				

*Abbreviations*: *N1* Number of persons with clinical examination, *N2* Number of persons with parasitological examination, *mf* microfilaria, *nod* nodule, *CMFL* community microfilarial load (microfilariae/skin snip), *nd* not done

### History of mass treatment with ivermectin

**Fig. 2:**
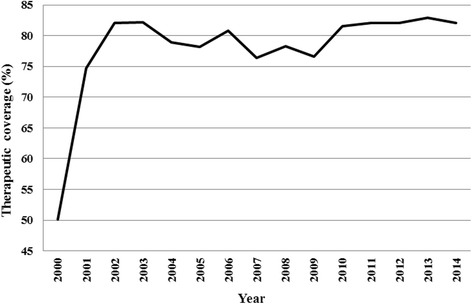
Therapeutic coverage of community-directed treatment with ivermectin in the West Region between 2000 and 2014

The treatment started in 1996 in the region with the support of Carter Center and Lions Club International Foundation, as a community based treatment with an annual dose of ivermectin. However, according to the National Onchocerciasis Control Programme (NOCP), CDTI started in 2000 in the region with a progressive enrollment of communities [30]. So, these communities received at least 15 rounds of mass drug administration, respectively. The therapeutic coverage achieved calculated over the total population was only 50% in 2000 and, from 2001 it was at least 75% as illustrated in Fig. 2. During the last five years of treatment campaigns, the treatment coverage was above 80%.

### Study design and patients

A cross-sectional survey was conducted in May 2015 in 15 communities of the Bafang (2), Bandja (1), Bandjoun (3), Bangangté (2), Foumbot (3), Kékem (1) and Massangam (3) health districts, nine months after the previous annual ivermectin distribution. All individuals, either permanent residents or who had already lived for at least five years in the community and aged five years or more were eligible for this survey. All volunteers underwent clinical and parasitological examinations. In addition, individual adherence to ivermectin treatment was also assessed.

#### Clinical examination

Each participant was examined for skin disease and nodules. The searched cutaneous signs were depigmentation, onchodermatitis (acute, chronic and lichenified), hanging groin and skin atrophy. Participants were scored as ‘positive’ or ‘negative’ to each cutaneous sign. Nodule palpation was performed in a closed but well-illuminated room. Qualified and certified staff performed the palpation on partially disrobed participants while paying particular attention to bony prominences of the torso, iliac crests and upper trochanter of the femurs. Onchocercal nodules were identified clinically as mobile masses beneath the skin, firm and painless [31–33]. Results were ranked as ‘positive’ or ‘negative’, and if positive, the number and location of all palpable onchocercal nodules were recorded.

#### Parasitological examination

Immediately following the nodule palpation, two skin snips were taken from each posterior iliac crest using a 2 mm corneoscleral punch (Holth-type). The skin samples were immediately placed, separately, into wells of microtitration plates containing a sterile normal saline solution. After 24 h incubation at room temperature, the skin snips were removed and the fluid from each well was examined under low magnification (40×) by trained laboratory technicians [34]. For positive results, the microfilariae were counted and the individual microfilarial densities were expressed as the arithmetic mean number of microfilariae in the two skin snips (mf/ss).

#### Assessment of the individual adherence to ivermectin mass treatment

Individual adherence to ivermectin mass treatment was assessed by asking to each participant if he/she swallowed ivermectin tablets during each of the previous five years (including the last CDTI-campaign). Ivermectin tablets were presented to the participants to make sure that the interview was targeting the right treatment. Participant answers were recorded on an individual form as “yes”, “no” or cannot remember for each of the last five years.

### Data analysis

All relevant data (clinical signs, nodule, mf count, and adherence to ivermectin mass treatment) were recorded into a purpose-built Microsoft Access database and subsequently exported into STATA 13 for statistical analysis. All analyses were weighted proportionally to age and gender distribution in the population, according to 2015 national demographic data projections (Additional file 1: Tables S1-S3) [35]. Cutaneous signs, nodules and microfilaridermia prevalences were expressed as the proportion of infected or affected individuals with the 95% exact confidence interval (CI). When the microfilarial count was positive, the intensity of infection was computed as the geometric mean and its 95% CI. The community microfilarial load (CMFL) is defined as the geometric mean number of microfilariae per skin snip (mf/ss) among adults aged 20 years or more. It was calculated using a log (x + 1) transformation $\left(\mathrm{CMFL}={\mathrm{e}}^{\frac{1}{\mathrm{N}}\sum \ln \left(\mathrm{x}+1\right)}-1\right)$ where x is the individual microfilarial density, and N the total number of individuals aged 20 years and above. The therapeutic coverages were calculated using the total number of participants in each community. Frequencies were compared using Chi-square tests. Mean intensities of infection were compared between age and gender subgroups using ANOVA with *F*-tests or *t*-tests. A *P*-value < 0.05 was considered as statistically significant.

## Results

In the seven HD, 2058 individuals were examined among whom 55.2% were women. The mean age was 28.4 ± 22.2 years and, higher in women 30.1 ± 22.4 than in men 26.3 ± 21.9 (unpaired Student’s *t* = 3.80, *P* < 0.001).

### Prevalence and intensity of infection

The weighted prevalence of microfilaridermia was 5.5% (95% CI: 4.6–6.7) in the whole group and varied from 4.7% in women to 6.5% in men (*χ* ^2^ = 3.0, *df* = 1, *P* = 0.08). The weighted prevalence of nodule presence was 2.1% (95% CI: 1.6–2.8) and varied from 2.6% in women to 4.8% in men (*χ* ^2^ = 2.0, *df* = 1, *P* = 0.16). The weighted prevalence of cutaneous signs presence was 1.7% (95% CI: 1.3–2.1) and varied from 1.1% in men to 2.2% in women (*χ* ^2^ = 3.6, *df* = 1, *P* = 0.058). Among microfilaridermia carriers, the geometric mean was 3.2 mf/ss (SD: 4.3) and varied from 3.0 mf/ss (SD: 4.9) in female to 3.5 mf/ss (SD: 3.9) in male (*t* = 1.13, *df* = 121, *P* = 0.26). Among nodule carriers, the geometric mean was 1.3 nodules per carrier (SD: 1.5) with no variation between males 1.3 (SD: 1.5) and females 1.3 (SD: 1.5) (*t* = -0.13, *df* = 69, *P* = 0.90).

Regarding age groups, the weighted prevalences of microfilaridermia varied respectively from 4.0% in 5–9 years old to 11.6% in 40–49 years old, with significant differences among age groups (*χ* ^2^ = 20.3, *df* = 6, *P* = 0.002). The presence of nodules increased with age, with weighted prevalences from 0.2% in 10–19 years old to 8.2% in 50–59 years old (*χ* ^2^ = 68.3, *df* = 6, *P* < 0.001). For cutaneous signs, there was a large difference between < 40 years and ≥40 (0.4% *vs* 10.8%; *χ* ^2^ = 145.3, *df* = 1, *P* < 0.001). The geometric mean of microfilaridermia among positive cases was higher in 20–29 years-old participants and decreased with age (ANOVA: *F*_(6, 116)_ = 2.32, *P* = 0.04), while there was no difference among age groups for nodule geometric means (ANOVA: *F*_(6, 64)_ = 0.95, *P* = 0.46) (Table 2). In the 30 children aged under 10 years we examined in Makouopsap, the weighted prevalences were 49.9% (95% CI: 32.0–67.9) for microfilaridermia and 13.3% (95% CI: 1.1–25.5) for nodule presence, respectively. In this age group, 15 out of 19 positive children overall for microfilaridermia and 4 out of 8 carrying nodules came from Makoupsap.

**Table 2:** Weighted prevalence of microfilaridermia, nodules and cutaneous signs presence according to gender and age groups

	*N*	Parasitological examination			Clinical examination				
		mf + (*n*)	Weighted mf prevalence (%)^a^	Geometric mean of mf in carriers (mf/ss ± SD)	Nodule + (*n*)	Weighted nodule prevalence (%)^a^	Geometric mean of nodule in carriers ± SD	cut + (*n*)	Weighted cut prevalence (%)^a^
Sex									
Female	1136	57	4.7	3.0 ± 4.9	29	1.7	1.3 ± 1.5	56	2.2
Male	922	68	6.5	3.5 ± 3.9	44	2.6	1.3 ± 1.5	24	1.1
Total	2058	125	5.5	3.2 ± 4.3	73	2.1	1.3 ± 1.5	80	1.7
Age categories (years)									
5–9	472	19	4.0	4.7 ± 4.7	8	1.7	1.2 ± 1.4	1	0.2
10–19	609	27	4.4	2.5 ± 4.8	1	0.2	1.0 ± (-)^b^	0	0
20–29	156	16	10.1	6.9 ± 2.9	5	3.2	1.2 ± 1.4	1	0.7
30–39	173	13	7.5	1.5 ± 2.4	14	8.0	1.4 ± 1.6	6	3.4
40–49	180	21	11.6	3.6 ± 5.1	11	6.1	1.3 ± 1.5	13	7.2
50–59	147	12	8.3	1.4 ± 2.4	12	8.2	1.0 ± 1.0	16	10.8
60+	297	15	5.1	2.8 ± 4.4	20	6.6	1.4 ± 1.8	41	14.0
Total	2034	123	5.5	3.2 ± 4.3	71	2.1	1.3 ± 1.5	78	1.7

*Abbreviations*: *N* number examined, *Mf + (n)* number with microfilaria positive skin snip, *Nod + (n)* number of nodule carriers, *Cut + (n)* number with cutaneous sign, *SD* standard deviation

^a^In each gender category, the indices were adjusted on age; In each age category, the indices were adjusted on sex; in the total populations, indices were adjusted on sex and age

^b^Standard deviation not calculated as the number of observations is 1

**Fig. 3:**
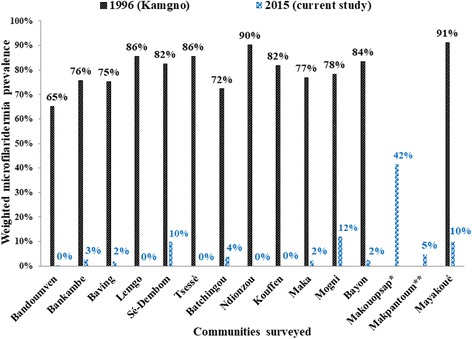
Comparison of weighted microfilaridermia at baseline in 1996 and in follow-up survey in 2015 in each surveyed community. *Community was not surveyed in 1996 but in 2011; **Community was not surveyed in 1996 nor in 2011

**Fig. 4:**
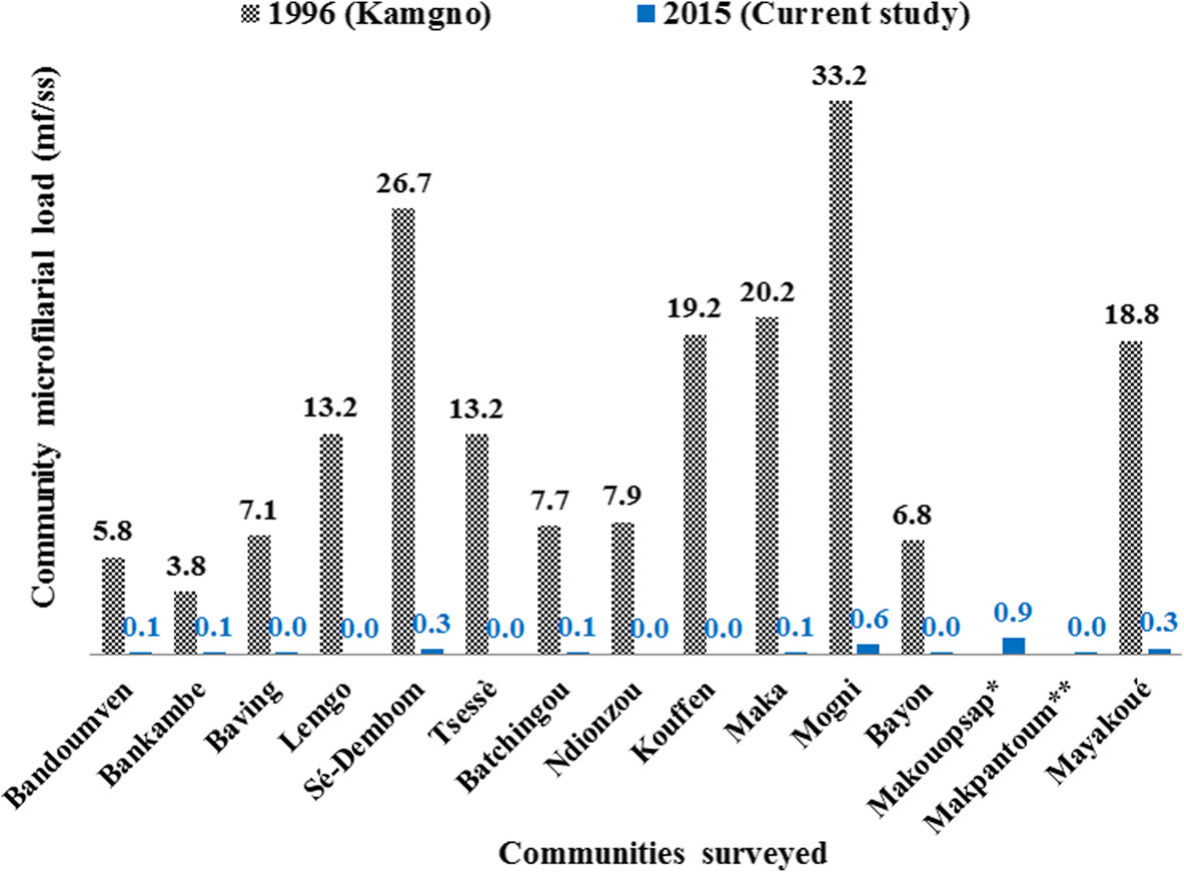
Comparison of community microfilaria load (CMFL) at baseline in 1996 and in follow-up survey in 2015 in each surveyed community. *Community was not surveyed in 1996 but in 2011; **Community was not surveyed in 1996 nor in 2011. *Abbreviations*: mf, microfilaria; ss, skin snip

In surveyed communities, the weighted prevalences of microfilaridermia varied from 0% (Lemgo, Tsesse, Ndionzou, Kouffen) to 41.6% (Makouopsap), with 11 (73.3%) communities having less than 5%. Except the community Makouopsap which had a prevalence of 41.6%, all the surveyed communities were below 15%. A significant reduction in microfilaridermia prevalence was observed (84.7–100%) in all the 13 communities, as compared to baseline data (Fig. 3). The CMFL in all the surveyed communities also significantly dropped by 98–100%, from 3.75–33.16 mf/ss in 1996 to 0–0.94 mf/ss in 2015 (Fig. 4). The weighted prevalences of nodule presence varied from 0% (Bandoumven, Baving, Ndionzou) to 14.4% (Makouopsap), with 14 (93.3%) communities having less than 5%. For cutaneous signs presence, the weighted prevalences varied from 0% to 13.3%, with 13 (86.7%) communities having less than 5% (Table 3). Onchocercal skin lesions were more frequent in two communities (Baving and Ndionzou) where lower limb depigmentation and chronic onchodermatitis were found (Table 3).

**Table 3:** Weighted prevalence of microfilaridermia, nodules and cutaneous signs presence in each surveyed community

			Parasitological examination			Clinical examination				
Health district	Community	*N*	mf + (*n*)	Weighted mf prevalence (%)^a^	Geometric mean of mf in carriers (mf/ss ± SD	Nod + (*n*)	Weighted nodule prevalence (%)^a^	Geometric mean of nodule in carriers ± SD	cut + (*n*)	Weighted cut prevalence (%)^a^
Bafang	Bandoumven	125	1	0.3	22.0 ± (-)^b^	0	0	–	7	1.7
	Bankambe	203	9	2.6	1.3 ± 2.9	11	2.9	1.3 ± 1.4	0	0
Bandja	Baving	136	3	1.8	1.7 ± 1.3	0	0	–	25	9.1
Bandjoun	Lemgo	75	0	0.0	–	3	1.1	1.0 ± 1.0	0	0
	Se Dembom	173	17	9.8	5.2 ± 4.2	1	0.2	1.0 ± (-)^b^	1	0.5
	Tsesse	123	0	0.0	–	1	0.4	1.0 ± (-)^b^	0	0
Bangangté	Batchingou	139	6	3.7	2.9 ± 2.7	10	2.7	1.3 ± 1.8	2	0.6
	Ndionzou	141	0	0.0	–	0	0	–	43	13.3
Foumbot	Kouffen	107	0	0.0	–	5	2.5	1.9 ± 1.8	0	0
	Maka	207	6	2.2	0.9 ± 2.1	2	0.9	1.0 ± 1.0	0	0
	Mogni	96	12	12.0	4.0 ± 3.3	6	3.9	1.4 ± 1.8	0	0
Kékem	Bayon	231	6	2.3	2.3 ± 2.8	3	0.5	1.0 ± 1.0	0	0
Massangam	Makouopsap	123	53	41.6	3.7 ± 5.4	26	14.4	1.3 ± 1.4	2	1.1
	Makpantoum	114	5	4.7	1.3 ± 1.9	3	2.4	1.0 ± 1.0	0	0
	Mayakoue	65	7	9.8	5.3 ± 3.9	2	2.6	1.4 ± 1.6	0	0
Total		2058	125	5.5	3.2 ± 4.3	73	2.1	1.3 ± 1.5	80	24.5

*Abbreviations*: *N* number examined, *Mf + (n)* number with microfilaria positive skin snip, *Nod + (n)* number of nodule carriers, *Cut + (n)* number with cutaneous sign, *SD* standard deviation

^a^In each gender category, the indices were adjusted on age; In each age category, the indices were adjusted on sex; in the total populations, indices were adjusted on sex and age

^b^Standard deviation not calculated as the number of observations is 1

**Fig. 5:**
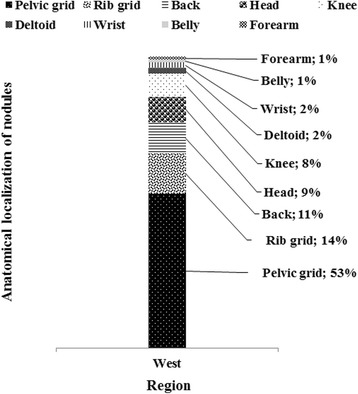
Distribution of nodules per anatomical localization in carriers

Most palpable nodules were localized in the pelvic grid, the chest, the head and the knee, with half of them found in the pelvic grid (Fig. 5).

### Adherence to ivermectin mass treatments in surveyed communities

**Fig. 6:**
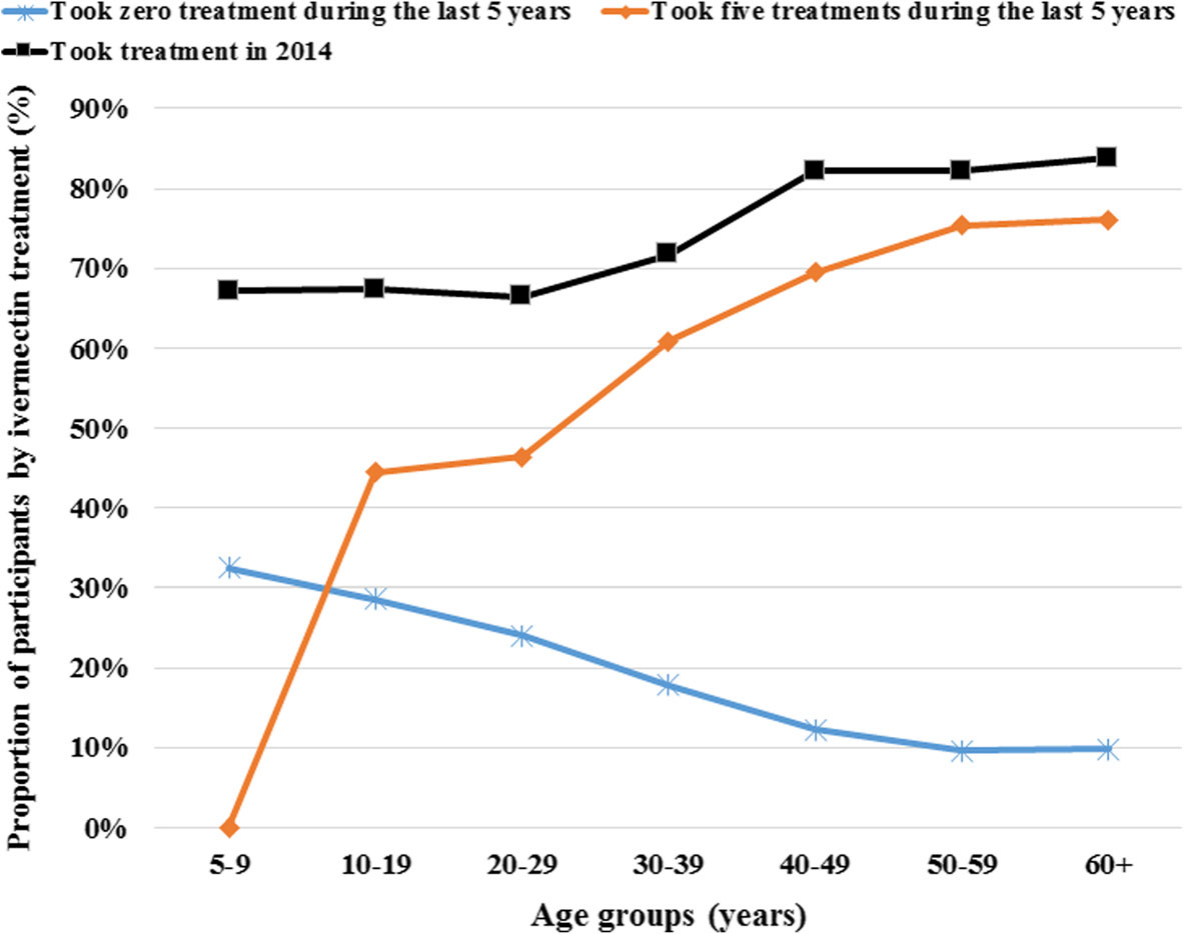
Therapeutic adherence to ivermectin treatment of participants per age groups

**Fig. 7:**
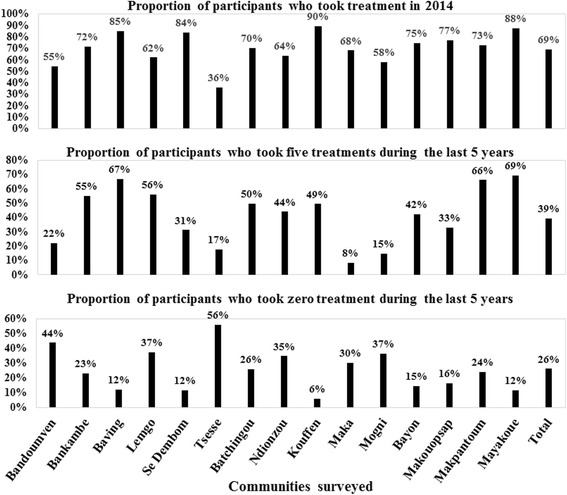
Therapeutic adherence to ivermectin treatment of participants in surveyed communities

The overall weighted therapeutic coverage in 2014 was 69.4% (95% CI: 67.4–71.4), with important variations across communities ranging from 35.9% (95% CI: 27.3–44.5) in Tsesse (Bandjoun HD) to 89.7% (95% CI: 83.8–95.5) in Kouffen (Foumbot HD). Only 39.3% (95% CI: 27.6–50.9) of participants declared to have swallowed ivermectin tablets each year during the past five years, ranging from 8.3% (95% CI: 4.9–11.7) in Maka (Foumbot HD) to 69.3% (95% CI: 57.2–81.4) in Mayakoue (Massangam HD). The reported adherence to treatment increased significantly with age since more than 70% of participants aged 40 years and above declared having taken the treatment each year during the last five years. More than 82% reported to have taken the treatment in 2014. More than one out of four participants (26.1%; 95% CI: 23.9–28.3) declared that they had not taken the treatment during the last five years. These non-adherent participants are mainly found at Bandoumven (Bafang HD), Lemgo and Tsesse (Bandjoun HD), Ndionzou (Bangangte HD), Maka and Mogni (Foumbot HD) with at least 30% in each community who declared not having taken the treatment (Figs. 6 and 7). The microfilaridermia prevalence was not, in general, associated with treatment adherence (*χ* ^2^ = 3.8, *df* = 5, *P* = 0.58), even in Makouopsap where the endemicity level was the highest (*χ* ^2^ = 3.0, *df* = 5, *P* = 0.70). The geometric mean of microfilaria among carriers was not associated with treatment adherence (ANOVA: *F*_(5, 117)_ = 2.18, *P* = 0.06).

## Discussion

The objective of the present survey was to assess the progress made towards the elimination of onchocerciasis in the West CDTI project by assessing unevaluated communities where the highest endemicity level were found in 1996, before the launch of control measures. This assessment was based on previous APOC’s conceptual framework for onchocerciasis elimination as it was conducted before the publication of the new 2016 WHO revised guidelines on stopping mass drug administration (MDA) and verifying elimination which advocate xenomonitoring and serology.

In 2015, after more than 15 years of ivermectin mass treatment, we observed a great reduction in microfilaridermia prevalence in all the 13 communities with baseline endemicity data, but still above the expected level at Makouopsap (Massangam HD) (≤ 20% for a pretreatment endemicity ≥ 80% assuming a therapeutic coverage of 65%) [26, 36]. In line with the observed decrease in the mf prevalences, the intensity of infection had considerably decreased in 2015, with CMFL below 1 mf/ss in all communities, even at Makouopsap.

The Makouopsap focus is close to the Mbam river, a fast-flowing river which also crossed the Bafia HD (Centre Region) where recent surveys found a microfilaridermia weighted prevalence as high as 57% in the community Ngongol [37]. Blackfly breeding sites located along the river contribute to maintain high vector densities and continuing transmission as it was previously demonstrated for first-line communities [38]. As also observed in some foci of the Bafia HD, the Makouopsap focus had the highest prevalence of microfilaridermia and nodule observed in less than 10-year-old children, born after the launch of the programme. This is a strong evidence of ongoing active transmission which could later be confirmed by OV-16 serology as recommended by the new WHO guidelines [27]. A joined innovative strategy (semi-annual ivermectin treatment, vector control) between the West and the Centre CDTI-projects, led by the National Onchocerciasis Control Programme (NOCP) should be implemented to definitively tackle the persistence of the disease across the bordering districts. Walker et al. [39] highlighted the potential importance of vector control in high-transmission settings as a complementary intervention strategy.

In the other surveyed communities, there is an encouraging progress towards the elimination of the disease with a less than 5% weighted microfilaridermia prevalence in 11/14 communities, however not enough to initiate a decrease in ivermectin distribution according to the new WHO guidelines [27]. As underlined in the guidelines, the skin snip sensitivity is low especially in newly infected person and after several ivermectin rounds which considerably reduce the microfilarial load. It is possible that the true prevalences are higher than those observed in this study as also mentioned by Bottomley et al. [40] who found that this sensitivity increases with the number of skin snips taken. Therefore, the NOCP, the communities and the different stakeholders involved in the control programme should be encouraged to improve and sustain their efforts.

The progress observed is threatened by many factors like (i) the vector burden, (ii) the low adherence to ivermectin treatment especially in younger persons, and (iii) the presence of the disease in children. We have not performed entomological surveys, but as the epidemiological situation has not changed at Makouopsap, we can also assume that the entomological situation remained unchanged too, especially as no vector control measure was implemented. We can rely on results reported by Katabarwa et al. [29, 41] in 2011 with an annual biting rate of 125,360 and an annual transmission potential of 310 in that locality. Considering the potential flight range of blackflies which can reach 500 km as observed in the reinvasion of some countries after vector control measures in West Africa [42], the focus of Makoupsap can be the source of reinfestation or maintenance of infection in the entire West Region and the neighboring Regions. Only 39% of the participants declared having taken five treatments during the last five years. Such low adherence to treatment is also a threat to the elimination of the disease. At the same time, about 26% of the participants did not take a treatment during that period. The low adherence observed in the less than 30-years group, not feeling the necessity to take a drug for a disease that they do not experience in the day-to-day life, constitutes an important hindrance in the success of the programme. With the observed adherence, the likelihood for the programme to achieve elimination of the disease by 2025 is quite low. Even though this survey was conducted nine months after the previous treatment, we believe that our results regarding the therapeutic coverage and the adherence are reliable because ivermectin tablets are special, by both their physical presentation (small and white) and their delivery strategy. So, the likelihood to confuse them with other drugs or interventions is quite low. Previous studies have reported accurate recall of populations when compared to the data from CDTI collected in treatment registry [43–45]. The presence of the disease in less than 10-year-old children is another threat as it means that the adult worms they carried, still have a lifespan of at least 5 to 10 years, constituting a source of reinfestation.

The oldest participants have more nodules than their youngest counterparts due to their prolonged exposure to the bites of blackflies, meanwhile their adherence to treatment could explain their lowest intensity of infection. This adherence is probably motivated by their awareness about the consequences of the disease.

The localization of nodules was comparable to previous studies [31–33, 37]. Onchocercal skin disease was more frequent in two communities (Baving and Ndionzou) where lower limb depigmentation, acute and chronic onchodermatitis were found.

## Conclusions

After more than 15 years of CDTI, except at Makouopsap, there is an important progress towards the elimination of onchocerciasis in the communities surveyed as part of this study. Additional efforts should be made to improve the CDTI adherence in all communities to maintain the achievement or greatly reduce the prevalence and the microfilarial densities to eliminate this debilitating disease. Innovative strategy like semi-annual ivermectin treatment plus a vector control strategy or the adjunction of a vector control strategy to the current annual treatment should be implemented at least in the bordering districts of the Centre and West Regions. Similar alternative interventions should also be implemented in other part of the country where the high prevalences of the disease persist to curb its progression in the sight of its elimination. Further studies targeting community therapeutic coverage, CDTI process and entomological studies in the Massangam district would provide better insights into our understanding of the persistence of the disease in that health district.

## Additional file

Additional file 1: Table S1. National demographic projections in 2015. **Table S2.** National demographic projections in 2015 for eligible persons. **Table S3.** Summary of national demographic projections in 2015 in eligible persons. (DOCX 26 kb)

## Abbreviations

APOC: African Programme for Onchocerciasis Control
ARES-CCD: Académie de Recherche et d’Enseignement Supérieur-Commission de la Coopération au Développement, Belgique
CDD: Community-directed distributors
CDTI: Community-directed treatment with ivermectin
CI: Confidence interval
CMFL: Community microfilarial load
CRFilMT: Centre for Research on Filariasis and other Tropical Diseases, Cameroon
HD: Health district
MDA: Mass drug administration
mf/ss: Microfilariae per skin snip
NOCP: National Onchocerciasis Control Programme, Cameroon
WHO: World Health Organization
