# Supplementary material for: Important progress towards elimination of onchocerciasis in the West Region of Cameroon
Source: Parasit Vectors. 2017 Aug 3;10:373. doi: 10.1186/s13071-017-2301-7 (PMC5543544; doi:10.1186/s13071-017-2301-7)
Supplement: Additional file 1: Table S1. — National demographic projections in 2015. Table S2. National demographic projections in 2015 for eligible persons. Table S3. Summary of national demographic projections in 2015 in eligible persons. (DOCX 26 kb) [file 13071_2017_2301_MOESM1_ESM.docx]

**Additional file 1. Demographic projections used for weighting**

**Table S1.** National demographic projections in 2015

|  | | | | | | | | |
| --- | --- | --- | --- | --- | --- | --- | --- | --- |
|  |  |  |  |  |  |  |  |  |
| **Age groups (years)** | **Sex** | | **Total Population** |  | **Age groups (years)** | **Sex** | | **Total Population** |
|  | **Male** | **Female** |  |  |  | **Male** | **Female** |  |
| **CAMEROON** | **10 955 014** | **11 224 693** | **22 179 707** |  | **CAMEROON** | **49.39%** | **50.61%** | **100.00%** |
| **0-4** | 1 864 628 | 1 819 548 | **3 684 176** |  | **0-4** | **8.41%** | **8.20%** | **16.61%** |
| **5-9** | 1 564 106 | 1 514 013 | **3 078 119** |  | **5-9** | **7.05%** | **6.83%** | **13.88%** |
| **10-14** | 1 362 549 | 1 301 532 | **2 664 081** |  | **10-14** | **6.14%** | **5.87%** | **12.01%** |
| **15-19** | 1 243 799 | 1 300 550 | **2 544 349** |  | **15-19** | **5.61%** | **5.86%** | **11.47%** |
| **20-24** | 1 004 919 | 1 154 854 | **2 159 773** |  | **20-24** | **4.53%** | **5.21%** | **9.74%** |
| **25-29** | 831 650 | 945 812 | **1 777 462** |  | **25-29** | **3.75%** | **4.26%** | **8.01%** |
| **30-34** | 679 747 | 715 211 | **1 394 958** |  | **30-34** | **3.06%** | **3.22%** | **6.29%** |
| **35-39** | 531 645 | 562 280 | **1 093 925** |  | **35-39** | **2.40%** | **2.54%** | **4.93%** |
| **40-44** | 446 601 | 458 866 | **905 467** |  | **40-44** | **2.01%** | **2.07%** | **4.08%** |
| **45-49** | 368 807 | 368 575 | **737 382** |  | **45-49** | **1.66%** | **1.66%** | **3.32%** |
| **50-54** | 292 041 | 284 363 | **576 404** |  | **50-54** | **1.32%** | **1.28%** | **2.60%** |
| **55-59** | 216 900 | 203 020 | **419 920** |  | **55-59** | **0.98%** | **0.92%** | **1.89%** |
| **60-64** | 170 894 | 174 328 | **345 222** |  | **60-64** | **0.77%** | **0.79%** | **1.56%** |
| **65-69** | 143 761 | 154 200 | **297 961** |  | **65-69** | **0.65%** | **0.70%** | **1.34%** |
| **70-74** | 104 543 | 116 306 | **220 849** |  | **70-74** | **0.47%** | **0.52%** | **1.00%** |
| **75-79** | 60 567 | 66 302 | **126 869** |  | **75-79** | **0.27%** | **0.30%** | **0.57%** |
| **80-84** | 34 956 | 42 704 | **77 660** |  | **80-84** | **0.16%** | **0.19%** | **0.35%** |
| **85+** | 32 901 | 42 229 | **75 130** |  | **85+** | **0.15%** | **0.19%** | **0.34%** |

**Table S2.** National demographic projections in 2015 for eligible persons

|  | | | | | | | | |
| --- | --- | --- | --- | --- | --- | --- | --- | --- |
|  |  |  |  |  |  |  |  |  |
| **Age groups (years)** | **Sex** | | **Total Population** |  | **Age groups (years)** | **Sex** | | **Total Population** |
|  | **Male** | **Female** |  |  |  | **Male** | **Female** |  |
| **CAMEROON** | **9 090 386** | **9 405 145** | **18 495 531** |  | **CAMEROON** | **49.15%** | **50.85%** | **100.00%** |
| **5-9** | 1 564 106 | 1 514 013 | **3 078 119** |  | **5-9** | **8.46%** | **8.19%** | **16.64%** |
| **10-14** | 1 362 549 | 1 301 532 | **2 664 081** |  | **10-14** | **7.37%** | **7.04%** | **14.40%** |
| **15-19** | 1 243 799 | 1 300 550 | **2 544 349** |  | **15-19** | **6.72%** | **7.03%** | **13.76%** |
| **20-24** | 1 004 919 | 1 154 854 | **2 159 773** |  | **20-24** | **5.43%** | **6.24%** | **11.68%** |
| **25-29** | 831 650 | 945 812 | **1 777 462** |  | **25-29** | **4.50%** | **5.11%** | **9.61%** |
| **30-34** | 679 747 | 715 211 | **1 394 958** |  | **30-34** | **3.68%** | **3.87%** | **7.54%** |
| **35-39** | 531 645 | 562 280 | **1 093 925** |  | **35-39** | **2.87%** | **3.04%** | **5.91%** |
| **40-44** | 446 601 | 458 866 | **905 467** |  | **40-44** | **2.41%** | **2.48%** | **4.90%** |
| **45-49** | 368 807 | 368 575 | **737 382** |  | **45-49** | **1.99%** | **1.99%** | **3.99%** |
| **50-54** | 292 041 | 284 363 | **576 404** |  | **50-54** | **1.58%** | **1.54%** | **3.12%** |
| **55-59** | 216 900 | 203 020 | **419 920** |  | **55-59** | **1.17%** | **1.10%** | **2.27%** |
| **60-64** | 170 894 | 174 328 | **345 222** |  | **60-64** | **0.92%** | **0.94%** | **1.87%** |
| **65-69** | 143 761 | 154 200 | **297 961** |  | **65-69** | **0.78%** | **0.83%** | **1.61%** |
| **70-74** | 104 543 | 116 306 | **220 849** |  | **70-74** | **0.57%** | **0.63%** | **1.19%** |
| **75-79** | 60 567 | 66 302 | **126 869** |  | **75-79** | **0.33%** | **0.36%** | **0.69%** |
| **80-84** | 34 956 | 42 704 | **77 660** |  | **80-84** | **0.19%** | **0.23%** | **0.42%** |
| **85+** | 32 901 | 42 229 | **75 130** |  | **85+** | **0.18%** | **0.23%** | **0.41%** |

**Table S3**. Summary of national demographic projections in 2015 in eligible persons

| Age groups (years) | Sex | | Total population (%) |
| --- | --- | --- | --- |
|  | Male (%) | Female (%) |  |
| 5-9 | 8.46 | 8.19 | 16.64 |
| 10-19 | 14.09 | 14.07 | 28.16 |
| 20-29 | 9.93 | 11.36 | 21.29 |
| 30-39 | 6.55 | 6.91 | 13.46 |
| 40-49 | 4.41 | 4.47 | 8.88 |
| 50-59 | 2.75 | 2.64 | 5.39 |
| 60+ | 2.96 | 3.22 | 6.18 |
| Total | 49.15 | 50.85 | 100 |
